# A Cross-Sectional Study on Benzene Exposure in Pediatric Age and Parental Smoking Habits at Home

**DOI:** 10.3390/ijerph17155469

**Published:** 2020-07-29

**Authors:** Arianna Antonucci, Matteo Vitali, Stefano Martellucci, Vincenzo Mattei, Carmela Protano

**Affiliations:** 1 Department of Public Health and Infectious Diseases, “Sapienza” University of Rome, 00185 Rome, Italy; matteo.vitali@uniroma1.it (M.V.); carmela.protano@uniroma1.it (C.P.); 2 Biomedicine and Advanced Technologies Rieti Center, “Sabina Universitas”, 02100 Rieti, Italy; s.martellucci@sabinauniversitas.it (S.M.); v.mattei@sabinauniversitas.it (V.M.)

**Keywords:** benzene, exposure, environmental tobacco smoke, children, parents, smoking, summer, winter, home smoking habits

## Abstract

After the introduction of the smoke-free legislation, household smoking has become the major source of environmental tobacco smoke (ETS) exposure for children. In our previous research, we found a strong association between urinary unmodified benzene (u-UB) levels and passive smoking exposure related to the home smoking policies (HSP). The aim of the study is to further investigate the impacts of several factors on ETS-exposure in childhood by using u-UB as tobacco-related carcinogen biomarker of exposure. Two cross-sectional studies were performed on the same target population of our previous research, in summer and winter season of the years 2017 and 2018, respectively. A questionnaire and a head space–solid phase micro-extraction/gas chromatography-mass spectrometry (HS-SPME/GC-MS) analytical method were used as investigative procedures. The improvement found in smoking habits, when compared to our previous surveys, reduced the levels of u-UB in children. However, significant differences related to the high number of smokers and smoked cigarettes, in total and at home, still persist. These differences are more relevant in the winter season. Finally, the only effective way for making homes completely smokefree is to develop public health policies for encouraging people to quit or drastically reduce smoking.

## 1. Introduction

Tobacco smoke is a complex mixture of thousands of compounds, found in particulate and vapour phases, which are proved to be very persistent in indoor environments (air, dust, and surfaces) far beyond the period of active smoking [1,2,3,4]. For some years now, environmental tobacco smoke (ETS), which includes second-hand smoke (SHS) [5,6,7] and third-hand smoke (THS) [8,9,10,11,12] and constitutes the main sources of exposure for nonsmokers to tobacco smoke toxicants, has been indicated as remarkable cause of many lung and cardiovascular diseases [13,14], as well as of different types of cancer [15,16,17]. The health risk of greatest concern to humans from long-term ETS exposure is carcinogenesis [18,19,20,21]. Specifically, low-level of exposure to benzene, the most significant human carcinogen component [22] of cigarette smoke mixture, was found to be responsible for acute myelogenous leukemia [23] and other lymphohematopoietic malignancies [24]. Several studies show that children represent a population at special risk for the adverse health effects of passive smoking [25,26,27,28,29,30,31], while there are no data indicating that low levels of children exposure to ETS are harmless. Indeed, specific epidemiological studies suggest that environmental benzene exposure in early life increases the risk of developing leukemia and other blood disorders in childhood [32,33,34,35,36]. Moreover, given the long latency of cancer diseases, much research should be conducted to assess the links between the risk of certain types of cancer during adulthood and ETS exposure during childhood.

ETS exposure occurs in indoor environments when tobacco products are smoked, mainly in homes where smokers live, but also in vehicles and public places. However, after the introduction of the smoking ban in public places, household environment has become the main source of exposure to ETS for children [37,38,39]. Accordingly, many studies established that benzene uptake in children is mainly related to involuntary cigarette smoke inhalation occurring in household environments [40]. In recent years and in relation to these issues, our studies have been focused on the contribution of smoking cohabitant(s)’ behaviors in children exposure to household airborne benzene. In particular, our previous research suggests that (a) urinary unmodified benzene (u-UB) is an effective exposure marker for benzene in children [29,41], (b) there is a strong association between u-UB concentrations in children and ETS exposure levels, and (c) u-UB levels are related to the smoking policy adopted at home (HSP), especially when urine samples are collected at the end of the day and in children living in areas at low traffic density. In a cross-sectional study carried out about ten years ago among children of central Italy [42] we found significant differences in u-UB levels between ETS-exposed and ETS-unexposed children, and with respect to the HSP of the children’s cohabitants. Specifically, the excretion of u-UB significantly increased in parallel to the enhancement of ETS exposure related to the non-compliance with the smoking ban at home. This finding was further confirmed by the use of u-cotinine, the best biomarker of active and passive smoking exposure [43]. In the study, we did not consider the influence of some factors to predict differences in benzene excretion in the studied population, such as the number of cohabitant smokers and their cigarettes consumption. We did not even take account the benzene uptake variation in different seasons that might occur due to the differences both in air recycling of homes and in the use of heating systems. In a recent study [44], the Authors observed a seasonal variation of the outdoor BTEX (benzene, toluene, ethylbenzene and xylenes) concentration (winter > spring > autumn > summer) and identified the vehicular exhaust as the primary source. Anyway, specific high levels of benzene indicated an irregular emission source of benzene other than traffic-related emissions.

Thus, the primary objective of the present study was to further investigate the impacts of both the HSPs of cohabitant smoker(s) and the sampling season on ETS exposure in childhood by the use of u-UB as tobacco-related carcinogen biomarker of exposure to ETS. The same geographic area and target population of our previous research [42] were investigated for monitoring possible changes in smoking habits of cohabitant smoker(s) at a distance of time and then assessing the differences related to u-UB excretion. In addition, summer and winter seasons were chosen to investigate possible seasonal influences in u-UB levels.

## 2. Materials and Methods

### 2.1. Study Design and Population

Two cross-sectional studies, in the summer and winter seasons, were performed among a large sample of 5–11 years-old children (in total 369 and 293 participants, respectively), recruited from primary district schools located in the same countries of central Italy (province of Rieti) of our previous study [42]. The low urbanization rate characterizing the selected sites, that can be considered “low rate of land use” areas as well-described previously [45], was chosen because useful to reduce as much as possible the interference of airborne benzene due to motor vehicle traffic on children’s u-UB levels. The two monitoring campaigns were conducted in the academic years 2017–2018 (the term-time, which in Italy begins in September and ends in June of the following year), during two typical weekdays. Specifically, the sample collection was performed on Wednesday evening and delivered to the research team on Thursday morning. We selected these days for evaluating the usual exposure of participants during the week, and far from the weekend when the exposure significantly changes (no school, peculiar activities such as visiting relatives, day out, etc.).

The study included the analytical quantification of u-UB in urine samples and the data processing of an ad-hoc questionnaire, specifically designed to investigate the possible factors associated with ETS exposure in childhood. The questionnaire was elaborated “ad hoc”, developed and administered in Italian language. The full version of the questionnaire is reported in the Supplementary File S1. All the questions were designed on the basis of the scientific literature [46,47,48] and on previous experience of the research group. Before its administration, the questionnaire was pilot tested by interviewing face-to-face 10 families with children 5–11 years old. The pilot study was performed to perfect the questionnaire and the last version was self-administered to 20 other parents that confirmed they understood the questions. All the subjects involved in the pilot study did not participate to the present study.

In particular, questions were elaborated to collect information on the socio-demographic characteristics of the children’s families and to elicit surrogate exposure data, for example information about the number of smokers who live with the child, their smoking habits and precautions taken by at-home smokers. Time–activity surveys were also used to determine the amount of time spent in household environments. Before agreeing to participate, all students and their parents received information about the aims and plans of the research other than the modalities to compile the questionnaire and to collect and store urine samples. Before starting, the study protocol and all the documents necessary to participate were submitted and approved by the local Ethical Committee (Policlinico Umberto I, “Sapienza” University of Rome; protocol code 2894). Informed consent and personal data processing authorization were signed by both parents of each participant and by older children (9–11 years old). Then, all data obtained from questionnaires and biological samples were treated anonymously only for scientific purposes.

### 2.2. Data Collection

Each participant provided one sample of urine, collected in a benzene-free polypropylene bottle just before going to sleep and immediately stored in the domestic refrigerator at about 4 °C. In the morning, biological samples and the fulfilled documents (informed consent for parents and for children aged ≥9 years, the personal data processing form and the ad-hoc questionnaire) were gathered at the belonging schools and delivered to the research team. Urine samples were placed into refrigerated containers, transported to laboratory and immediately partitioned into glass vials as follows: 20 mL glass vials containing 3 g of dried NaCl were filled with 14 mL of urine sample and sealed with holes aluminum caps with Teflon-lined septa. Then the vials were coded and frozen at −20 °C, until analysis.

### 2.3. Analytical Determination

Headspace solid phase micro-extraction (SPME) technique was used to extract u-UB from urine samples, while analytical determinations were performed by gas chromatography-mass spectrometry (GC-MS). Methodology and analytical performances (linearity, precision, recovery, accuracy, detection and quantitation limit values) are reported in details in a previous paper [49]. Briefly, the frozen samples were thawed out at room temperature and maintained at 30 °C for 60 min in an aluminum block device (VWR, Milan, Italy); then, analytes were sampled by an 85 μm carboxen/polydimethylsiloxane SPME fiber (Supelco, Bellafonte, PA, USA) for 15 min and injected into the GC-MS system (Trace GC ULTRA coupled to Focus DSQ MS, Thermo Fisher Scientific, Waltham, MA, USA) operating in the SIM mode. Matrix effect was compensated by preparing blanks, calibration and working standards with urine from children not exposed to ETS and flushed with nitrogen gas to further decrease u-UB concentration. Calculated six-point linear plots and benzene-d_6_ as internal standard were used for the quantification of u-UB. The limit of quantification (LOQ) was calculated as the arithmetic mean of sample blank values obtained by the analysis of 15 consecutive blanks plus 10 times its standard deviation (SD). Samples with urinary benzene concentrations <LOQ (7.4 ng/L) were excluded from statistical analysis.

### 2.4. Covariates and Statistical Elaboration

Based on questionnaire replies, we examined different characteristics concerning personal life and family that may affect benzene exposure of each participant. Seasonal concentration variations of u-UB were also examined.

In descriptive statistics, we reported absolute and relative frequencies for categorical variables and arithmetic mean ± SD for continuous variables (age and the number of cigarettes smoked in total and at home).

The investigated variables were coded and added in a database, specifically elaborated for statistical purposes. Specifically, related codes were assigned in accordance with the following information obtained via questionnaire: gender (0 = male; 1 = female); ponderal status according to sex-specific body mass index-for-age growth charts produced by the Centers for Disease Control and Prevention (CDC) [50] (1 = Underweight, 2 = Normal weight, 3 = Overweight, 4 = Obesity); educational level of each parent according to the Organization for Economic Co-operation and Development [51] (1 = basic, ≤9 years; 2 = upper secondary, ≤14 years; 3 = tertiary/higher, ≥17 years); house density measured as square footage × inhabitant (0 = high density, ≤30 m^2^/inhab.; 1 = low density, >30 m^2^/inhab.) [42]; house floor (0 = ground floor, 1 = upper floors) [42]; home heating devices (0 = methane or electric heaters, 1 = oil or wood heaters); time spent indoor a day (0 = hours < 17, 1 = hours ≥ 17); daily extra-curricular activities (0 = no activity, 1 = at least one daily activity); ETS exposure status in the domestic environment (0 = no; 1 = yes, when is present at least one cohabitant smoker). Since previous studies found that overweight and obesity can significantly influence the urinary excretion of biological indicators [52], we grouped the ponderal status as “underweight and healthy weight” or “overweight and obese” for comparing the category “overweight and obese” versus the others.

Then, in order to investigate the influence of domestic ETS exposure on benzene levels, only for children exposed to ETS, the following information were insert into our database: home smoking policy (0 = total smoke ban, when cohabitant smoker(s) do not smoke at home; 1 = partial smoking ban, when cohabitant smoker(s) smoke at home only when the child is out; 2 = no smoking ban, when cohabitant smoker(s) smoke at home also when the child is present); number of cohabitant smoker(s) (0 = only one smoker; 1 = more than one smoker); cohabitant smoker (0 = mother; 1 = father; 2 = others); number of cigarettes smoked in total (0 = cigarettes ≤ 20, 1 = cigarettes > 20); number of cigarettes smoked at home (0 = cigarettes ≤ 10, 1 = cigarettes > 10).

Statistical analyses were performed using IBM SPSS Statistics software package (version 25, IBM Corp., Armonk, NY, USA). Measured u-UB levels, grouped by ETS exposure status (0 or 1, if no-one cohabitant smokes or is present at least one cohabitant smoker), were checked for the assumption of normal distributions with the Kolmogorov-Smirnov test. Then, statistical elaboration was performed using parametric methods (*t*-test for independent variables, one-way analysis of variance (ANOVA) with Bonferroni post-hoc tests and Pearson’s correlation) on ln-values. The significance level chosen for all statistical tests was *p* < 0.05 (two tailed). Pairwise deletion was used for missing values.

## 3. Results

In total, 369 and 293 healthy children participated to the summer and winter biomonitoring campaigns, respectively (about 73% and 70% participation rate for the summer and winter surveys). Few of these were excluded for an inadequate urine sample. Then, about 5% of this population, not of Caucasian origin, was excluded from statistical analysis to avoid variations in tobacco exposure biomarker concentrations induced by ethnicity of target population, widely recognized as potential confounder factor [53,54]. At last, 313 (with slight female preponderance, 52.1%) and 263 (with a slight male preponderance, 55.1%) samples were available for the data processing in the two seasons. Table 1 and Table 2 summarize the main features of the study population of summer and winter surveys, respectively.

For both seasons, the average age was about 9 years. Then, the similar average age and gender distributions for the sub-population of children exposed to ETS were recorded in each season. Only about 50% of the investigated population had a healthy weight. As stated in many other surveys [55], children spent most of their time indoors both in summer (82.7%) and in winter (90.1%) and did not practice any extra-curricular activities in the sampling day. Within the family, the mother has generally a higher level of education than the father. Similar characteristics concerning exposure to ETS emerged in the two monitoring campaigns. About 40% of the participants was exposed to ETS. The mother was the most frequent smoker among the smoking cohabitants (59.8% in summer, 56.0% in winter). Based on the HSPs adopted by cohabitant smoker(s), a relatively small percentage of exposed children (18.9% in summer and 23.0% in winter) were living in homes without any smoking ban. In fact, only a small proportion of cohabitant smokers (24 units in summer and 23 in winter) declared to smoke at home at least one cigarette in presence of their children. Then, even a smaller group (17.3% in summer and 18.0% in winter) was living with one or more smokers who do not observe a total smoking ban at home. Moreover, the number of total cigarettes smoked at home, not considering the presence of children, were generally very small (i.e., 61.2% of cohabitant smokers in summer and 65.2% in winter affirmed to smoke less than 5 cigarettes inside the home).

Table 3 shows the levels of u-UB concentrations according to some characteristics of all participants, of their parents and of their homes.

In both seasons, when considering the entire population, most of the differences in u-UB concentrations related to the investigated variables were not statistically significant. The gender variable in summer and the paternal education variable in winter were the only exceptions (*p* = 0.001 and *p* = 0.026, respectively). In the summer survey, the u-UB difference between ETS exposed and not exposed children (mean values 35.62 ng L^−1^ and 38.12 ng L^−1^, respectively) was not significant (*p* = 0.572). In winter, despite the same home smoking behaviors of summer season were adopted, the difference between the u-UB levels of exposed (mean value = 58.98 ng L^−1^) and not exposed (mean value = 41.06 ng L^−1^) children was significant (*p* = 0.005). Then, in order to assess the influence of ETS-exposure modes on the u-UB levels, we limited further analysis to the participants exposed to ETS only. u-UB levels referring to the HSPs and smoking habits of cohabitant smoker(s) are reported in Table 4.

In the current biomonitoring campaigns, we detected very low urinary benzene levels compared with those of ten years ago. In both seasons, the differences of u-UB levels according the ponderal status variable were not significant, despite the related concentrations were still higher for children overweight or obese.

In the summer survey, the excretion of u-UB increased with respect to the number of both the cohabitant smokers (mean = 31.83 ng L^−1^ for only one smoker, mean = 45.68 ng L^−1^ when the smokers were more than one; *p* = 0.009) and the total smoked cigarettes (from 33.29 ng L^−1^ for less or equal to 20 cigarettes to 52.15 ng L^−1^ for more than 20 cigarettes; *p* = 0.037). With respect to the HSP, there was no significant difference of u-UB levels between children living with adults not smoking inside the home (total smoking ban, 33.23 ng L^−1^) and those living with adults smoking inside home only when children are out (partial smoking ban, 36.22 ng L^−1^). Greater differences, even if not yet statistically significant, were observed only when no smoking restriction was applied at home (mean = 45.59 ng L^−1^ for cohabitant(s) smoking inside the home even when children are in). The most significant difference was observed when the number of cigarettes smoked at home was considered, with or without the presence of the child (mean = 34.74 ng L^−1^ for children living in house where cohabitants smoke less than 10 cigarettes inside; mean = 65.82 ng L^−1^ when the smoked cigarettes were more than 10). The difference between groups was significant, with *p* value of 0.007.

In the winter survey we observed trends similar to summer ones in u-UB levels. As in summer, the excretion of u-UB significantly increased in parallel to the enhancement of ETS exposure at home, mostly related to the number of cohabitant smokers (from a mean value of 54.25 ng L^−1^ when there is only one smoker among cohabitants to 68.04 ng L^−1^ for children living with more than one smoker; *p* = 0.046) and the number of smoked cigarettes (from 56.85–65.93 ng L^−1^ when the smoked cigarettes increased above the number of 20; *p* = 0.021). Also, the differences among u-UB levels with respect to the home smoking policy were slightly greater than in summer, even if not yet significant. The mean values of related concentrations were distributed as follows: 54.84 ng L^−1^ when cohabitant(s) not smoke inside the home, 55.61 ng L^−1^ when cohabitant(s) smoke inside the home only when children are out and 69.95 ng L^−1^ when cohabitant(s) smoke inside the home even when children are in. Again, the most significant difference was observed with respect to the number of cigarettes smoked at home, with or without the presence of the child (mean = 54.52 ng L^−1^ for children living in house where cohabitant(s) smoke less than 10 cigarettes inside; mean = 84.90 ng L^−1^ when the cigarettes smoked at home were more than 10; *p* = 0.001).

Finally, in both seasons, when there was only one smoker at home, the exposure was significantly greater when the smoker was the mother (41.41 ng L^−1^ in summer and 63.15 ng L^−1^ in winter when smokes only the mother; 28.98 ng L^−1^ in summer and 48.42 ng L^−1^ in winter when smokes only the father). Actually, maternal smoking is usually looked as the largest source of ETS for the child because of the close proximity to the mother during early life.

Table 5 reports the u-UB concentrations according to some home characteristics and the practice of an extra-curricular sport activity, comparing ETS exposed and not-exposed children.

Although no significant differences have been found relating the characteristics of domestic environments and the practice of after-school activities in the sampling day, the trend in u-UB concentrations was consistent with the occurrence of household exposure to ETS. For example, the differences in u-UB levels between children living in high density houses and those living in low density houses were about seven times the same difference referred to not ETS-exposed children (differences equal to 5.95 ng L^−1^ vs. 0.83 ng L^−1^, respectively). This is what should have been expected given the strong correlation between airborne benzene intake and ETS exposure at home. Besides, our results suggest that ETS-exposure affected u-UB more than the floor on which the house is located. In fact, the exposure to environmental benzene related to vehicular exhaust fumes should be higher when living on ground floor, as confirmed by data related to children not ETS-exposed. Actually, it resulted higher in children living in upper floors (38.61 ng L^−1^ vs. 33.25 ng L^−1^). In relation to the time activity, among ETS-exposed children, those who did not practice extra activities and then spent more time in domestic environments where smokers live, had the highest levels of u-UB (40.85 ng L^−1^ vs. 34.11 ng L^−1^). The investigated differences in u-UB excretion were greater in the winter season (Table 3), as it is typically characterized by discontinuous air supply and longer permanence at home [56]. Despite this, the correlations between benzene exposure and household ETS exposure with respect to the residential home environment features and to the performed extra-curricular activities were less congruent then in summer (Table 4). Effectively, in this season there is less control and understanding of such contributes to the household ETS exposure, mainly due to the use of fuels such as coal, liquid petroleum gas, or wood for space heating, which provide a relevant source of exposure to environmental benzene in the home [56,57]. Even if not significant u-UB differences were found in relation to the heating system, the influence of such factor on the urinary levels of benzene was confirmed by the highest values found for children living in homes heated by fireplaces or combustion stoves.

The relationship between u-UB concentrations and age was evaluated by the use of Pearson’s correlation (Table 6).

As shown in Table 6, in summer season, u-B concentration significantly increased with age, while in winter season this positive association was found in children exposed to ETS.

## 4. Discussion

This study was conducted in order to further evaluate the influence of smoking habits of cohabitants on children exposure to ETS in domestic environments. The findings are useful to add new evidence on passive smoking exposure during pediatric age.

The first relevant result is related to the frequency of children exposed to ETS: about 40% of the participants were exposed to ETS in domestic environments. This finding is in line with the percentage of children exposed to passive smoking estimated worldwide [58] and with the percentage of Italian adult smokers (20%) [59].

The current ETS exposure rates are very low compared with those found for the same study population about ten years ago [42]. Our previous results showed that about 50% of exposed children lived with at least one adult smoking inside the home even when child is present. As a consequence, we observed high levels of u-UB among the study population, with concentration values of some hundreds of ng L^−1^, other than large differences between ETS-exposed and unexposed children (median values 359.50 vs. 92.50 ng L^−1^). Thus, the results of the present study demonstrated better practice adopted on regard the home smoking policy by cohabiting smokers compared to the past. Similar findings were found also in a study performed in Germany: the proportion of children who are exposed to ETS at home has significantly declined over the years [60].

According to the information recovered by the questionnaire, over 60% of them adopted a total smoking ban in domestic environments but, at the same time, those who did not observe this responsible policy still smoked few cigarettes at home. Schoolchildren u-UB levels in the summer and winter seasons were of the same order of magnitude and, as expected, given the more responsible home smoking policy adopted by cohabitant smokers, were generally smaller than those found in our previous survey performed in the same study area. In both seasons, we observed a significantly reduction of the differences in u-UB levels between ETS exposed and unexposed children other than the actual values itself. Very small differences of u-UB levels were observed inside the exposed children category between children whose parents do not smoke at home and those that smoke at home only when children are out (categories of total and partial smoking ban). We observed yet a slight increasing in mean values of u-UB when no ban on smoking at home was observed, mainly because it was never complete and it was referred to only few cigarettes. In both seasons, the degree of exposure increased in direct proportion to the intensity of the parents’ smoking habits as it depended especially on the number of smokers and the amount of smoked cigarettes, total and at home, with or without the presence of the children. This result is in line to that reported previously [61,62]. Wang et al. [61] demonstrated that the magnitude of exposure to passive smoking during paediatric age is correlated with the smoking behaviors of the caregiver. Likewise, Park [62] demonstrated that living with a smoking parent was linked with exposure to carcinogens, and that smoking at home increase ETS exposure.

In summer, u-UB levels also agreed with the characteristics of the indoor spaces where exposure occurred (described by m^2^ per inhabitant and the house floor) and with the duration of exposure (accounted for by the performed extra-curricular activities), even if no significant differences were observed. Besides, in summer we found a significant age-dependency of UB, in line with other previous studies evaluated urinary levels of trace elements in children [63,64]. This relationship is probably linked to the growth of children, but present only in summer season. Thus, it is plausible that the significant positive correlation between u-UB and age is not due to differences in metabolism and excretion processes, but rather due to differences in attitudes and activities of higher children; it should be remarkable to further study this association. The same positive association was found in children exposed to ETS during the winter season; it is presumable that cohabiting smoker tend to protect to a greater extend younger children.

The greatest correlation between u-UB levels and domestic ETS exposure was observed in the winter season, because of the limited air exchanges and the longer time spent at home, that are typical of the coldest season of the year, especially in this territory characterized by high hills and a severe winter. Several studies [65,66,67] evidenced that increasing ventilation, as usual in the summer season, can reduce indirect exposures to tobacco smoke to very low levels. Instead, no correlation between u-UB levels and characteristic of household spaces or time spent at home were found in the winter season due to the employment of combustion heaters, widely qualified as the major exposure sources to environmental benzene [68].

Our results agree with previous researches [69] that reported indoor ETS concentration to be dependent on the number of cigarettes smoked in that location, the size of the room and the air exchange. In addition to this, the current findings show that when the smoke load increases (large numbers of smoked cigarettes), toxicants from SHS that persist on carpets, furnishings and walls as well as on clothes, skin and hairs of the smoker, are gradually released back into the air, even if smoking occurs at times or in rooms when no children are present.

This kind of tobacco pollution, more persistent in domestic environments and more difficult to remove by venting the rooms, poses an additional risk of exposure for children (THS). In these cases, the respect of good rules of conduct on household smoking generally may reduce exposure levels but does not help to bring the exposure values to the order of the unexposed children, adversely affecting the good behavior adopted on regard the home smoking policy. Then, the heavy smokers not smoking at home but still carrying toxic tobacco substances on their clothes and body, only partially protect their children from household-ETS since they pollute their house even when they do not smoke inside [8,12].

Some limitations of the present study should be noted. First, the cross-sectional study gives a snapshot of the population, not followed in the time. Second, we collected for each participant just one spot urine sample at the end of the day, and not different samples or a 24-h urine collection. Thus, we did not assess possible changes in excretion of urinary u-benzene during the day or its total excretion in a day. However, urinary u-benzene levels provide an estimation of a child’s recent exposure. Third, no airborne monitoring of living environments has been carried out during the two biomonitoring campaigns. Therefore, it would be interesting to propose again the study by planning the sampling for several consecutive days, with the chance of monitoring possible variations in biomarker excretion within the 24 h and establishing a correlation between the airborne benzene level in a specific environment and its intake

## 5. Conclusions

Trends in children exposure to ETS emerging from the present study indicate that levels of exposure have declined significantly since our previous research (from 47.7% to about 20% for both seasons of children living with at least one adult who smoked inside the home even when children were present). The stricter smoking ban has had an impact on the reduction of exposure in children, confirming that changes in the home smoking policies, by increasing the smoking restrictions in homes where children reside, play a key role in the observed decline of u-UB concentrations among children. In addition, while the recent findings suggest that the HSPs improving over the years is essential to reduce the levels of u-UB, significant differences still persist in its excretion, not depending from the home smoking policy but in relation to the number of smoker and smoked cigarettes, in total and at home. This is due to the major contribution of THS to the household ETS. Since ventilation or limiting smoking to certain areas do not provide sufficient protection in these cases, the only effective way of making enclosed spaces completely ETS-free and reduce the harm of its exposure on children is to develop public health policies for encouraging people to quit or reduce smoking.

Although the promising findings presented here, the evidence concerning the adverse health effects of ETS exposure for children are strong enough to promote active intervention for the achievement of 100% smoke-free household environments. Moreover, because there is no safe level of exposure to ETS and parental smoking in the home was identified as the highest source of ETS exposure among children, this represents the only effective strategy to provide an acceptable level of protection of children from the dangers of ETS exposure.

Then, multidisciplinary educational programs could be the effective strategy in promoting smoke-free environment in the home, by informing parents about the risks of SHS and THS exposures and how to protect their families from ETS harm.

The significant reduction of u-UB levels achieved among the children population studied ten years ago lead to think that our project should be an example of such strategy: the participation and the assumption of an active role in the study occurred some time ago have been making the parents and teachers aware of the importance to reduce ETS in indoor environments to actively protect children.

## Figures and Tables

**Table 1:** Characteristics of the study population participating to the summer biomonitoring campaign.

Variable			Descriptives in % if Not Stated Otherwise (*n*)		
			Unexposed to ETS ^1,2^	Exposed to ETS ^1,2^	All Participants
			(*n* = 183)	(*n* = 127)	(*n* = 313)
Age ^3^			Mean 8.67 (Standard Deviation 1.54)	Mean 8.69 (Standard Deviation 1.40)	Mean 8.68 (Standard Deviation 1.48)
Gender ^3^		Male	49.2 (*n* = 90)	46.5 (*n* = 59)	47.9 (*n* = 150)
		Female	50.8 (*n* = 93)	53.5 (*n* = 68)	52.1 (*n* = 163)
Ponderal status according to BMI ^4^		Under weight	8.8 (*n* = 16)	6.3 (*n* = 8)	7.7 (*n* = 24)
		Healthy weight	53.0 (*n* = 97)	48.0 (*n* = 61)	50.5 (*n* = 158)
		Overweight	18.0 (*n* = 33)	15.8 (*n* = 20)	16.9 (*n* = 53)
		Obese	7.6 (*n* = 14)	17.3 (*n* = 22)	11.5 (*n* = 36)
		Unknown	12.6 (*n* = 23)	12.6 (*n* = 16)	13.4 (*n* = 42)
Maternal education (years)		Basic (≤9 years)	13.7 (*n* = 25)	19.7 (*n* = 25)	16.0 (*n* = 50)
		Upper secondary (≤14 years)	44.8 (*n* = 82)	52.8 (*n* = 67)	47.6 (*n* = 149)
		Tertiary/higher (≥17 years)	38.2 (*n* = 70)	26.0 (*n* = 33)	32.9 (*n* = 103)
		Unknown	3.3 (*n* = 6)	1.5 (*n* = 2)	3.5 (*n* = 11)
Paternal education (years)		Basic (≤9 years)	21.8 (*n* = 40)	32.3 (*n* = 41)	25.9 (*n* = 81)
		Upper secondary (≤14 years)	43.2 (*n* = 79)	49.6 (*n* = 63)	45.4 (*n* = 142)
		Tertiary/higher (≤17 years)	30.6 (*n* = 56)	15.0 (*n* = 19)	23.9 (*n* = 75)
		Unknown	4.4 (*n* = 8)	3.1 (*n* = 4)	4.8 (*n* = 15)
House density (m^2^/inhabitant)		<30	62.3 (*n* = 114)	70.9 (*n* = 90)	65.2 (*n* = 204)
		>30	32.8 (*n* = 60)	26.8 (*n* = 34)	30.0 (*n* = 94)
		Unknown	4.9 (*n* = 9)	2.3 (*n* = 3)	4.8 (*n* = 15)
House floor		0	20.2 (*n* = 37)	20.5 (*n* = 26)	20.1 (*n* = 63)
		≥1	78.1 (*n* = 143)	77.2 (*n* = 98)	77.0 (*n* = 241)
		Unknown	1.7 (*n* = 3)	2.3 (*n* = 3)	2.9 (*n* = 9)
Time spent indoor/daily		<17 h	13.7 (*n* = 25)	11.0 (*n* = 14)	12.5 (*n* = 39)
		≥17 h	82.5 (*n* = 151)	85.0 (*n* = 108)	82.7 (*n* = 259)
		Unknown	3.8 (*n* = 7)	4.0 (*n* = 5)	4.8 (*n* = 15)
Extra-curricular sport activities/day ^5^		No	58.5 (*n* = 107)	59.8 (*n* = 76)	59.1 (*n* = 185)
		Yes	40.4 (*n* = 74)	39.4 (*n* = 50)	39.6 (*n* = 124)
		Unknown	1.1 (*n* = 2)	0.8 (*n* = 1)	1.3 (*n* = 4)
		Cohabitant smoker	Mother	59.8 (*n* = 76)	
			Father	32.3 (*n* = 41)	
			Others	4.7 (*n* = 6)	
			Unknown	3.2 (*n* = 4)	
**Only exposed to ETS ^1^**		Home smoking policy	Total smoking ban	62.2 (*n* = 79)	
			Partial smoking ban	17.3 (*n* = 22)	
			no smoking ban	18.9 (*n* = 24)	
			Unknown	1.6 (*n* = 2)	
		Number of cohabitant smoker(s)	1	67.7 (*n* = 86)	
			>1	30.7 (*n* = 39)	
			Unknown	1.6 (*n* = 2)	
		Number of cigarettes smoked in total/daily		Mean 16.73	
				(Standard Deviation 10.45)	
		Number of cigarettes smoked at home/daily		Mean 2.47	
				(Standard Deviation 4.37)	

Note: ^1^—ETS: environmental tobacco smoke; ^2^—Unknown = 3; ^3^—Unknown = 0; ^4^—BMI: body mass index (weight in kg/height squared in m); ^5^—The sampling day.

**Table 2:** Characteristics of the study population participating to the winter biomonitoring campaign.

Variable		Descriptives in % If Not Stated Otherwise (*n*)		
		Unexposed to ETS ^1,2^	Exposed to ETS ^1,2^	All Participants
		(*n* = 156)	(*n* = 100)	(*n* = 263)
Age ^3^		Mean 8.67 (Standard Deviation 1.38)	Mean 9.22 (StandardDeviation 6.71)	Mean 8.89 (Standard Deviation 4.32)
Gender ^3^	Male	55.8 (*n* = 87)	54.0 (*n* = 54)	55.1 (*n* = 145)
	Female	44.2 (*n* = 69)	46.0 (*n* = 46)	44.9 (*n* = 118)
Ponderal status according to BMI ^4^	Under weight	7.1 (*n* = 11)	8.0 (*n* = 8)	7.2 (*n* = 19)
	Healthy weight	51.9 (*n* = 81)	42.0 (*n* = 42)	46.8 (*n* = 123)
	Overweight	12.8 (*n* = 20)	17.0 (*n* = 17)	15.2 (*n* = 40)
	Obese	12.2 (*n* = 19)	19.0 (*n* = 19)	14.5 (*n* = 38)
	Unknown	16.0 (*n* = 25)	14.0 (*n* = 14)	16.3 (*n* = 43)
Maternal education (years)	Basic (≤9 years)	14.1 (*n* = 22)	21.0 (*n* = 21)	16.4 (*n* = 43)
	Upper secondary (≤14 years)	48.1 (*n* = 75)	54.0 (*n* = 54)	49.8 (*n* = 131)
	Tertiary/higher (≥17 years)	33.9 (*n* = 53)	24.0 (*n* = 24)	30.4 (*n* = 80)
	Unknown	3.9 (*n* = 6)	1.0 (*n* = 1)	3.4 (*n* = 9)
Paternal education (years)	Basic (≤9 years)	18.6 (*n* = 29)	33.0 (*n* = 33)	24.0 (*n* = 63)
	Upper secondary (≤14 years)	50.6 (*n* = 79)	51.0 (*n* = 51)	50.6 (*n* = 133)
	Tertiary/higher (≤17 years)	26.9 (*n* = 42)	15.0 (*n* = 15)	22.4 (*n* = 59)
	Unknown	3.9 (*n* = 6)	1.0 (*n* = 1)	3.0 (*n* = 8)
House density (m^2^/inhabitant)	<30	57.0 (*n* = 89)	69.0 (*n* = 69)	61.6 (*n* = 162)
	>30	38.5 (*n* = 60)	25.0 (*n* = 25)	33.5 (*n* = 88)
	Unknown	4.5 (*n* = 7)	6.0 (*n* = 6)	4.9 (*n* = 13)
House floor	0	21.8 (*n* = 34)	20.0 (*n* = 20)	21.3 (*n* = 56)
	≥1	73.7 (*n* = 115)	74.0 (*n* = 74)	73.8 (*n* = 194)
	Unknown	4.5 (*n* = 7)	6.0 (*n* = 6)	4.9 (*n* = 13)
Domestic heating devices	Methane or electric heaters	75.0 (*n* = 117)	67.0 (*n* = 67)	68.8 (*n* = 181)
	Oil or wood heaters	23.1 (*n* = 36)	32.0 (*n* = 32)	27.4 (*n* = 72)
	Unknown	1.9 (*n* = 3)	1.0 (*n* = 1)	3.8 (*n* = 10)
Time spent indoor/daily	<17 h	2.6 (*n* = 4)	4.0 (*n* = 4)	3.0 (*n* = 8)
	≥17 h	92.3 (*n* = 144)	90.0 (*n* = 90)	90.1 (*n* = 237)
	Unknown	5.1 (*n* = 8)	6.0 (*n* = 6)	6.9 (*n* = 18)
Extra-curricular sport activities/day ^5^	No	62.2 (*n* = 97)	64.0 (*n* = 64)	63.1 (*n* = 166)
	Yes	35.9 (*n* = 56)	33.0 (*n* = 33)	34.6 (*n* = 91)
	Unknown	1.9 (*n* = 3)	3.0 (*n* = 3)	2.3 (*n* = 6)
	Cohabitant smoker	Mother	56.0 (*n* = 56)	
		Father	39.0 (*n* = 39)	
		Others	3.0 (*n* = 3)	
		Unknown	2.0 (*n* = 2)	
**Only exposed to ETS ^1^**	Home smoking policy	Total smoking ban	51.0 (*n* = 51)	
		Partial smoking ban	18.0 (*n* = 18)	
		No smoking ban	23.0 (*n* = 23)	
		Unknown	8.0 (*n* = 8)	
	Number of cohabitant smoker(s)	1	65.0 (N = 65)	
		>1	34.0 (N = 34)	
		Unknown	1.0 (N = 1)	
	Number of cigarettes smoked in total/daily		Mean 15.61	
			(Standard Deviation 9.01)	
	Number of cigarettes smoked at home/daily		Mean 2.68	
			(Standard Deviation 5.09)	

Note: ^1^—ETS: environmental tobacco smoke; ^2^—Unknown = 7; ^3^—Unknown = 0; ^4^—BMI: body mass index (weight in kg/height squared in m); ^5^—The sampling day.

**Table 3:** u-Benzene concentrations (ng L^−1^) according to some characteristics of participants, of their parents and of their living environments.

Variable		u-Benzene Concentration	*p*-Value	u-Benzene Concentration	*p*-Value
		Summer Season		Winter Season	
		Mean (Standard Deviation)		Mean (Standard Deviation)	
Gender	Male	41.13 (30.69)	0.001	48.64 (46.78)	0.743
	Female	31.09 (17.37)		47.58 (44.83)	
Ponderal status according to BMI ^1^	Under or healthy weight	34.88 (23.09)	0.433	42.38 (33.54)	0.090
	Overweight or obese	38.23 (31.15)		57.76 (60.78)	
Maternal education	Upper secondary or lower	35.96 (25.74)	0.862	50.20 (33.17)	0.245
	Tertiary/higher	35.74 (23.93)		43.24 (39.36)	
Paternal education	Upper secondary or lower	35.78 (25.05)	0.778	50.57 (47.81)	0.026
	Tertiary/higher	36.12 (25.61)		39.48 (36.49)	
House density (m^2^/habitant)	≤30	37.09 (26.37)	0.875	48.78 (42.89)	0.523
	>30	34.08 (21.47)		47.78 (50.16)	
House floor	0	34.89 (25.25)	0.683	43.96 (40.45)	0.582
	≥1	36.83 (25.25)		50.15 (48.20)	
Domestic heating devices	Methane or electric heaters	-	-	45.91 (41.57)	0.263
	Oil or wood heaters	-		51.87 (54.42)	
Extra-curricular sport activities/day ^2^	No	36.26 (28.58)	0.789	48.24 (45.67)	0.643
	Yes	37.23 (28.90)		50.40 (44.43)	
Exposed to ETS ^3^	Not exposed	35.62 (23.91)	0.572	41.06 (33.94)	0.005
	Exposed	38.12 (26.83)		58.99 (58.05)	

Note: ^1^—BMI: body mass index (weight in kg/height squared in m); ^2^—The sampling day; ^3^—ETS: environmental tobacco smoke.

**Table 4:** u-Benzene concentrations (ng L^−1^) according to home smoking habits of participants’ cohabitant smoker(s), just considering the group of children exposed to environmental tobacco smoke (ETS).

Variable		u-Benzene Concentration	*p*-Value	u-Benzene Concentration	*p*-Value
		Summer Season		Winter Season	
Gender	Male	42.05 (34.95)	0.074	60.78 (61.44)	0.707
	Female	31.48 (16.28)		57.51 (55.01)	
Ponderal status according to BMI ^1^	Under or healthy weight	34.01 (21.97)	0.166	41.56 (30.77)	0.075
	Overweight or obese	40.72 (36.11)		79.19 (77.55)	
Home smoking policy	Total smoking ban	33.23 (18.35)	0.383	54.84 (52.72)	0.361
	Partial smoking ban	36.22 (20.89)		55.61 (55.62)	
	No smoking ban	45.59 (45.19)		69.95 (69.99)	
Number of cohabitant smoker(s)	1	31.83 (18.53)	0.009	54.26 (47.66)	0.046
	>1	45.68 (37.61)		68.04 (73.97)	
Cohabitant smoker(s)	Mother	41.50 (43.45)	0.025	63.15 (54.97)	0.032
	Father	28.70 (14.37)		48.42 (42.69)	
Number of cigarettes smoked/daily	≤20	33.29 (30.22)	0.037	56.84 (61.30)	0.021
	>20	52.15 (40.49)		65.92 (46.37)	
Number of cigarettes smoked at home/daily	≤10	34.74 (29.17)	0.007	54.52 (50.87)	0.001
	>10	65.82 (54.68)		84.90 (87.01)	

Note: ^1^—BMI: body mass index (weight in kg/height squared in m).

**Table 5:** u-Benzene concentrations (ng L^−1^) according to home characteristics and the practice of an extra-curricular sport activity, comparing ETS exposed and not-exposed children.

Variable		u-Benzene Concentration	*p*-Value ^1^	u-Benzene Concentration	*p*-Value ^1^
		Summer Season		Winter Season	
		Exposed/Not-Exposed		Exposed/Not-Exposed	
House density (m^2^/inhabitant)	≤30	39.41 (31.09)/35.26 (23.24)	0.537	56.10 (51.73)/42.75 (35.71)	0.660
	>30	33.46 (18.64)/34.43 (21.19)		65.06 (71.03)/40.66 (32.66)	
House floor	0	33.25 (21.33)/36.05 (27.89)	0.456	56.41 (55.83)/39.55 (27.21)	0.332
	≥1	38.61 (37.00)/35.60 (23.15)		59.70 (59.94)/42.52 (36.42)	
Domestic heating devices	Methane or electric heaters Oil or wood heaters	-	-	55.67 (49.73)/40.43 (35.08)	0.321
				61.97 (71.78)/41.95 (31.20)	
Extra-curricular sport activities/day ^2^	No	40.85 (39.83)/33.62 (22.63)	0.444	57.21 (59.77)/39.13 (32.04)	0.756
	Yes	34.11 (22.23)/38.26(25.67)		59.09 (52.86)/42.94 (36.83)	

Note: ^1^—Referred to ETS-exposed children; ^2^—The sampling day.

**Table 6:** Pearson’s correlation between u-Benzene concentrations (ng L^−1^, expressed as natural log transformed data) and age for all participants and just considering the group of children exposed to ETS or number of cigarettes smoked total or home/day.

Season	Age	u-Benzene Concentration	
		Pearson’s Coefficient	*p*-Value
Summer	All participants	0.118	0.037
	Children exposed to ETS	0.075	0.400
Winter	All participants	0.080	0.200
	Children exposed to ETS	0.245	0.013

## Supplementary Materials

The following are available online at https://www.mdpi.com/1660-4601/17/15/5469/s1, File S1: Questionnaire in Italian language realized ad hoc and used for the research.
