# Supplementary material for: A Cross-Sectional Study on Benzene Exposure in Pediatric Age and Parental Smoking Habits at Home
Source: Int J Environ Res Public Health. 2020 Jul 29;17(15):5469. doi: 10.3390/ijerph17155469 (PMC7432498; doi:10.3390/ijerph17155469)
Supplement: Supplementary file 1 [file ijerph-17-05469-s001.pdf]

CODICE IDENTIFICATIVO (apporre etichetta) | | **DATA DI COMPILAZIONE** ____/____/______

Gentili genitori,

nell'ambito della nostra indagine desideriamo porVi alcune domande per ottenere informazioni generali e su alcune abitudini di vita del Vostro/a bambino/a e del Vostro nucleo familiare.
Queste informazioni ci consentiranno di avere un quadro generale dell'ambiente di vita dei bambini che partecipano alla ricerca. Per tale motivo, Vi ringraziamo fin da ora per la Vostra preziosa collaborazione.

INFORMAZIONI SUL BAMBINO PARTECIPANTE

SESSO	DATA DI NASCITA	PESO CORPOREO	ALTEZZA	NAZIONALITÀ
☐ Maschio<br>☐ Femmina	|_|_|/|_|_|/|_|_|_|_|	|_|_| Kg	|_|_|_| cm	☐ Italiana<br>☐ Altro (Specificare):________________________

Il bambino pranza abitualmente alla mensa scolastica?
☐ Sì ☐ No

Il bambino assume regolarmente farmaco/i?
☐ Sì ☐ No
Se Sì, indicare quale/i farmaco/i e per quale motivo____

____

Il bambino segue una dieta particolare?
☐ Sì ☐ No
Se Sì, indicare perché ☐ Allergia ☐ Intolleranza ☐ Sovrappeso ☐ Altro, Specificare____________________

Pensando agli ultimi 12 mesi, rispondere alle seguenti 5 domande

1 **Quante ore trascorre mediamente il bambino a casa e fuori casa ogni giorno?**
(Il totale delle ore deve essere pari a 24. Se il tempo è inferiore a un'ora, indicare i minuti: 10 minuti, 15 minuti, etc)

**In luoghi chiusi** *(Abitazione, scuola, case di parenti o amici, le ore in cui il bambino dorme)*	
**In luoghi aperti** *(Anche l'eventuale giardino di casa)*	
**In auto**	
**Su mezzi pubblici**	
**Totale ore**	**24**

2 **Il bambino pratica o ha praticato un'attività sportiva extrascolastica?**
☐ Sì ☐ No
Se Sì, specificare:

**Tipo/i di sport**<br>*(Se il bambino pratica più sport, indicarli uno per uno in ciascuna riga)*	**Luogo**<br>*A = all'aperto*<br>*C = al chiuso*	**Numero di volte a settimana**	**Minuti per ogni volta**	**Per quanti mesi durante l'anno**

3 **Il bambino pratica o ha praticato altre attività extrascolastiche?**
(Hobby, corsi di lingua straniera, catechismo, etc)
☐ Sì ☐ No

Se Sì, indicare quale/i e con quale frequenza____

____

4 **Che tipo di acqua beve abitualmente il bambino? Segnare anche più di una risposta**
☐ Acqua di rubinetto ☐ Acqua di rubinetto con caraffa filtrante ☐ Acqua minerale in bottiglia
☐ Acqua di rubinetto con apparecchio filtrante e gasatore ☐ Altro, specificare______________________

5 **Quanti bicchieri di acqua beve mediamente il bambino ogni giorno?**
☐ 1-3 bicchieri ☐ 4-6 bicchieri ☐ 7-9 bicchieri ☐ 10 o più bicchieri

Pensando alla giornata di raccolta del campione di urine, rispondere alle seguenti 7 domande

1 **Quante ore ha trascorso il bambino a casa e fuori casa?**
(Il totale delle ore deve essere pari a 24. Se il tempo è inferiore a un'ora, indicare i minuti: 10 minuti, 15 minuti, etc)

**In luoghi chiusi** *(Abitazione, scuola, case di parenti o amici, le ore in cui il bambino dorme)*	
**In luoghi aperti** *(Anche l'eventuale giardino di casa)*	
**In auto**	
**Su mezzi pubblici**	
**Totale ore**	**24**

2 **Il bambino pratica o ha praticato un'attività sportiva extrascolastica?**
□ Sì □ No
Se Sì, specificare:

**Tipo/i di sport** *(Se il bambino pratica più sport, indicarli uno per uno in ciascuna riga)*	**Luogo** *A = all'aperto C = al chiuso*	**Numero di volte a settimana**	**Minuti per ogni volta**	**Per quanti mesi durante l'anno**
	□ A □ C			
	□ A □ C			
	□ A □ C			

3 **Il bambino ha praticato altre attività extrascolastiche?**
(Hobby, corsi di lingua straniera, catechismo, etc)
□ Sì □ No

Se Sì, indicare quale/i____

____

4 **Che tipo di acqua ha bevuto il bambino nell'arco della giornata? Segnare anche più di una risposta**
□ Acqua di rubinetto □ Acqua di rubinetto con caraffa filtrante □ Acqua minerale in bottiglia
□ Acqua di rubinetto con apparecchio filtrante e gasatore □ Altro, specificare______________________________

5 **Quanti bicchieri di acqua ha bevuto il bambino ?**
□ 1-3 bicchieri □ 4-6 bicchieri □ 7-9 bicchieri □ 10 o più bicchieri

6 **Il bambino ha bevuto altre bevande nell'arco della giornata?**
(Ad esempio un bicchiere di succo di frutta, coca-cola, aranciata, etc)
□ Sì □ No

Se Sì, indicare quale/i e la quantità____________________________________

7 **Cosa ha mangiato il bambino nell'arco della giornata?**
Indicare gli alimenti consumati dal bambino durante la giornata, la quantità e il tipo di pentola utilizzata per gli alimenti cucinati

COLAZIONE<br>*Ad esempio, tazza di latte, succo di frutta, fette biscottate, merendina, etc*	
MERENDA MATTUTINA<br>*Ad esempio una merendina, una pizzetta, un panino, etc*<br>*<u>Se fatta a scuola, indicare solo scuola</u>*	
PRANZO<br>*Ad esempio, piatto di pasta, piatto di minestra, pesce, carne, uova, formaggi, verdura, frutta*<br>*<u>Se fatta a scuola, indicare solo scuola</u>*	
MERENDA POMERIDIANA<br>*Ad esempio una merendina, una pizzetta, un panino*<br>*<u>Se fatta a scuola, indicare solo scuola</u>*	
CENA<br>*Ad esempio, un piatto di pasta, un piatto di minestra, pesce, carne, uova, formaggi, verdura, frutta, dolce, etc*	

INFORMAZIONI SUI GENITORI DEL BAMBINO

	Nazionalità	Titolo di Studio					Professione						
		Nessuno	Elementare	3° media	Diploma	Laurea	casalinga/o	operaia/o	impiegata/o	dirigente	libero professionista	pensionato	in cerca di occupazione
Madre	□ Italiana<br>□ Altro<br>____________	□	□	□	□	□	□	□	□	□	□	□	□
Padre	□ Italiana<br>□ Altro<br>____________	□	□	□	□	□	□	□	□	□	□	□	□

INFORMAZIONI SULL'ABITAZIONE IN CUI VIVE IL BAMBINO

Tipo di abitazione in cui vive il bambino

☐ Casa indipendente ☐ Condominio ☐ Altro (Specificare____________________)

Indicare anche:

Municipio di residenza____________________________

Principale tipologia di riscaldamento dell'abitazione____________________

Altre forme di riscaldamento *(camini, stufe a gas, etc)* ☐ Sì ☐ No Se Sì, indicare quale/i____________

Piano dell'abitazione *(seminterrato, terra, 1°, etc)*____________________

Superficie in m² |__|__|__|

Numero di persone che vivono nell'abitazione *(compreso il bambino)* |__|__|

Numero di fumatori che vivono nell'abitazione *(indicare 0 se nessuno fuma)* |__|__|

Nel caso in cui il bambino viva con uno o più fumatori, indicare anche:

**Convivente fumatore del bambino** *(indicare chi: madre, padre, sorella, fratello, nonni, etc)*	**Numero di sigarette fumate, in media, ogni giorno in totale**	**Numero di sigarette fumate, in media, ogni giorno in casa** *(indicare 0 se non fuma in casa)*	**Numero di sigarette fumate, in media, ogni giorno in casa in presenza del bambino** *(indicare 0 se non fuma in presenza del bambino)*
	|__|__|	|__|__|	|__|__|
	|__|__|	|__|__|	|__|__|
	|__|__|	|__|__|	|__|__|
	|__|__|	|__|__|	|__|__|
	|__|__|	|__|__|	|__|__|

IL QUESTIONARIO È TERMINATO, GRAZIE PER LA COLLABORAZIONE
